# Characterization of Phytochemicals and Antioxidant Activities of Red Radish Brines during Lactic Acid Fermentation

**DOI:** 10.3390/molecules19079675

**Published:** 2014-07-07

**Authors:** Pu Jing, Li-Hua Song, Shan-Qi Shen, Shu-Juan Zhao, Jie Pang, Bing-Jun Qian

**Affiliations:** 1 Key Lab of Urban Agriculture (South), Research Center for Food Safety and Nutrition, Bor S. Luh Food Safety Research Center, School of Agriculture & Biology, Shanghai Jiao Tong University, Shanghai 200240, China; E-Mails: pjcolumbus@gmail.com (P.J.); lihuas@sjtu.edu.cn (L.-H.S.); ssq927@gmail.com (S.-Q.S.); zshj_1012@126.com (S.-J.Z.); 2 College of Food Science, Fujian Agriculture and Forestry University, Fuzhou 350002, China; E-Mail: pang3721941@163.com

**Keywords:** radish, brine, fermentation, phytochemicals, antioxidant

## Abstract

Red radish (*Raphanus* L.) pickles are popular appetizers or spices in Asian-style cuisine. However, tons of radish brines are generated as wastes from industrial radish pickle production. In this study, we evaluated the dynamic changes in colour properties, phenolics, anthocyanin profiles, phenolic acid composition, flavonoids, and antioxidant properties in radish brines during lactic acid fermentation. The results showed that five flavonoids detected were four anthocyanins and one kaempferol derivative, including pelargonidin-3-digluoside-5-glucoside derivatives acylated with *p*-coumaric acid, ferulic acid, *p*-coumaric and manolic acids, or ferulic and malonic acids. Amounts ranged from 15.5–19.3 µg/mL in total monomeric anthocyanins, and kaempferol-3,7-diglycoside (15–30 µg/mL). 4-Hydroxy-benzoic, gentisic, vanillic, syringic, *p*-coumaric, ferulic, sinapic and salicylic acids were detected in amounts that varied from 70.2–92.2 µg/mL, whereas the total phenolic content was 206–220 µg/mL. The change in colour of the brine was associated with the accumulation of lactic acid and anthocyanins. The ORAC and Fe^2+^ chelation capacity of radish brines generally decreased, whereas the reducing power measured as FRAP values was increased during the fermentation from day 5 to day 14. This study provided information on the phytochemicals and the antioxidative activities of red radish fermentation waste that might lead to further utilization as nutraceuticals or natural colorants.

## 1. Introduction

Anthocyanins are water-soluble vacuolar pigments found in most species in the plant kingdom, and are responsible for the red, purple, and blue colours of many fruits, vegetables, cereal grains, and flowers [1]. The uses of anthocyanins as colorants have gained prominence as a result of both legislative action and consumers’ concerns over the use of synthetic additives in foods. Radish anthocyanins have been found to possess high tinctorial power and considerable stability, being a useful alternative to FD&C Red No. 40 (allura red) [2], which is one of the most consumed colorants in the world. Radish anthocyanins have been widely applied as natural colorants due to their colour characteristics as well as health benefits, including antioxidant activities [3,4].

Previous studies have reported that the pigment contents in different radish cultivars varied from 5 to 53 mg/100 g FW [5], or 64–161 mg/100 g FW [6]. Radish pigments belong to the pelargonidin-type anthocyanins [7,8], differing in type and number of acyl groups according to cultivar or growing location [5,9,10,11,12]. Radishes have been also reported to have a high amount of total phenolics [13], including phenolic acids [14] and flavonoids, mainly in the form of kaempferol [15].

Fermented vegetables are traditional but still popular appetizers or spices in Asian-style cuisine. Modern pickle manufacturers typically apply direct vat cultures for their performance consistency and reliability, instead of traditional fermentation. Fermentation with yeast [16], fungi [17], and bacteria [18] could significantly enhance the releasable antioxidant properties, and increase total phenolic and phenolic acids, and anthocyanin contents in wheat bran and black soybean. Therefore, fermentations not only produce lactic acid to impact food taste and flavor, but also possibly release considerable amounts of water-soluble bioactive compounds into the water medium and thus enhance the antioxidant activities [19].

Red radish (*Raphanus* L.) pickles are one type of important fermented vegetables for their colorful and texture properties. Tons of this kind of pickle are annually produced for domestic consumption and for export. Considerable amounts of radish brine are generated as a waste, which represents a challenge for full utilization of radish brines. However, the changes in phytochemical compositions, colour properties, and antioxidant activities in the colour-rich brine produced during lactic acid fermentation of red radishes are still not clear. The present study was thus conducted to evaluate the dynamic changes of total phenolics, anthocyanin profiles, phenolic acid composition, flavonoids, and their antioxidant properties during lactic acid fermentation. Additionally the variation of the colour density and characteristics were evaluated. This study might provide information on the phytochemicals produced in the red radish fermentation process and their antioxidative activities, as well as colour properties and could be helpful for the further development of red radish fermentation byproducts as nutraceuticals or natural colorants.

## 2. Results and Discussion

### 2.1. Lactic Acid, pH and Colour Properties of Radish Brines

Table 1 shows that lactic acid concentration increased significantly to 4.8 and 5.7 g/L respectively on the 2nd and 5th day after fermentation with *Lactobacillus plantarum*. Further fermentation for an additional 4 or 9 days did not increase lactic acid levels significantly (*p* > 0.05). The accumulation of lactic acid contributed predominantly to the change in pH values that decreased significantly to approximate 3.4 in the initial two days of fermentation (*p* < 0.01). The pH dropped continually to approximate 2.2 at the end of the 14-day fermentation. Changes in colour of the samples occurred due to lactic acid accumulation and anthocyanin release in the radish brines (Table 1). Lightness value was 71.21 after 2-day fermentation and did not change significantly during following fermentation (*p* > 0.05). The chroma was monitored as an indicator of changes in colour saturation during fermentation. The chroma value changed from 0 to 54.4 on the 2nd day, and did not significantly change on the 5th and 9th day of fermentation until on the 14th day when the chroma was decreased to 42.69 (*p* < 0.05), suggesting that the loss of pigments occurred. The colour in brine appeared a red with a hue angel of approximate 23° after fermentation for 2 days and a slight increase to 26°–27° on the 5th, 9th or 14th day.

**Table 1:** pH, lactic acid, and colour properties of the radish brines during fermentation.

Days	Lactic Acid (mg/mL)	pH	Lightness	Chroma	Hue
0^§^	0 ± 0.00a	6.50 ± 0.04a	100 ± 0.00a	0 ± 0.00a	0 ± 0.00a
2	4.80 ± 0.14b	3.42 ± 0.03b	71.21 ± 1.23b	54.4 ± 0.63b	23.28 ± 1.22b
5	5.71 ± 0.34c	3.36 ± 0.03b	68.42 ± 1.75b	50.19 ± 2.28c	26.25 ± 1.34c
9	6.42 ± 0.27c	3.31 ± 0.04b	69.73 ± 0.70b	49.67 ± 1.33c	26.59 ± 0.41c
14	6.23 ± 0.34c	2.21 ± 0.03c	71.63 ± 3.41b	42.69 ± 3.12d	27.22 ± 0.74c

^§^ The NaCl solution was tested as the sample on the day 0. Data are expressed as mean ± standard deviation (*n* = 3). Within each column, means with the different letter are significantly different (*p* < 0.05).

### 2.2. Identification of Flavonoids in Brines

**Figure 1:**
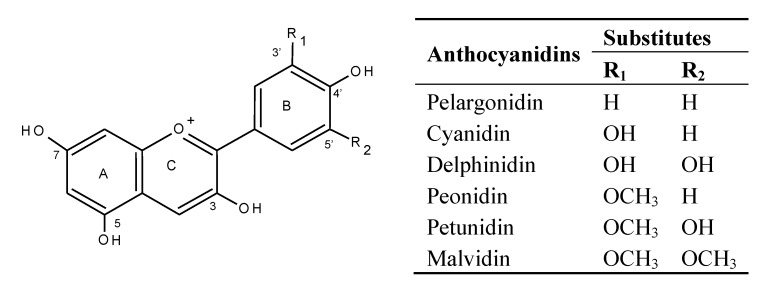
Structures of six common anthocyanidins occurring in Nature.

**Figure 2:**
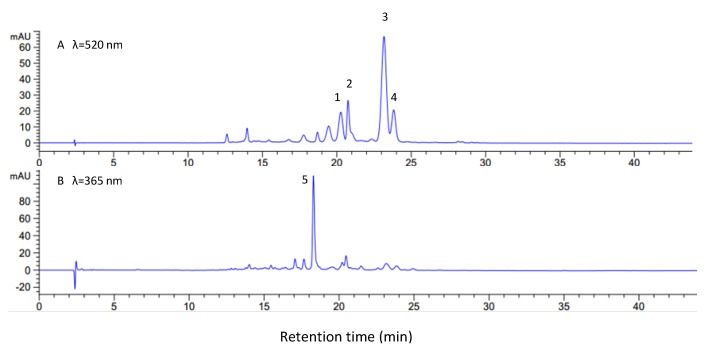
HPLC profiles of anthocyanins (**A**) and kaempferol-3,7-diglycoside (**B**) in radish brine.

Pelargonidin as one of six common anthocyanidins in Nature (Figure 1) was identified as the aglycone of anthocyanins in various red radish cultivars [7]. The individual anthocyanins in radish brines were identified by LC-MSn before quantification by HPLC profiles. There were five major peaks found in radish brines in Figure 2 at 210–600 nm. Among those, four peaks were anthocyanins, with a maximum visible wavelength of absorbance around 520 nm in Figure 2A. The four major anthocyanins were identified as the pelargonidin 3-diglucoside-5-glucoside acylated with *p*-coumaric (peak 1), ferulic (peak 2), *p*-coumaric and malonic (peak 3), ferulic and malonic acids (peak 4) based on fragmentation patterns of individual peaks in Table 2 and literature reports [20,21].

**Table 2:** Qualitative analyses of anthocyanins and kaempferol derivatives in radish brine after fermentation with *Lactobacillus plantarum*.

Peak	Compounds	λ_max_ (nm)	Fragments
1	Pg-3-(*p*-coumaroyl)diglu-5-glu	508	903 [M]^+^, 741 [M-glu]^+^, 433 [Pg+glu]^+^, 271 [Pg]^+^
2	Pg-3-(feruloyl)diglu-5-glu	510	933 [M]^+^, 771 [M-glu]^+^, 433 [Pg+glu]^+^, 271 [Pg]^+^
3	Pg-3-(*p*-coumaroyl)diglu-5-(malonyl)glu	514	989 [M]^+^, 741 [M-glu-mal]^+^, 519 [Pg+glu+mal]^+^, 271 [Pg]^+^
4	Pg-3-(feruloyl)diglu-5-(malonyl)glu	511	1019 [M]^+^, 771 [M-glu-mal]^+^, 519 [Pg+glu+mal]^+^, 271 [Pg]^+^
5	Kaempferol-3,7-(glucoside+rhamnoside)	365	593 [M]^−^, 447 [M-rhamnose]^−^, 431 [M-glu]^−^, 285 [kaemperol]^-^

Abbreviation: Pg, pelargonidin; diglu, diglucoside; glu, glucoside; mal, malonic acid.

Peak 5 in Figure 2B had a maximum wavelength of absorbance at 365 nm was identified as kaempferol-3,7-diglycoside with a glycosylation pattern of 3-glucoside-7-rhamoside or -3-rhamoside-7-glucoside (593 [M]^−^, 447 [M−rhamnose]^−^, 431 [M−glu]^−^, and 285 [kaemperol]^−^), which were reported as major flavonoids in radishes [15].

### 2.3. Change in Flavonoids in Brines during Fermentation

About 19 µg pelargonidin-3-glucoside equivalents (PGE)/mL monomeric anthocyanins were rapidly released into the brine after 2 days of fermentation (Table 3). No significant changes were observed in the concentration of total monomeric anthocyanins in radish brines during the subsequent fermentation from the 5th to the 9th day, suggesting that monomeric anthocyanins released from radish roots to brines, reached an equilibrium point during the initial two days of fermentation and maintained a consistent concentration until the 14th day, when the total monomeric anthocyanins slightly degraded to approximate 15 µg PGE/mL. The changes in individual anthocyanins as peak area percentages of the four anthocyanins are also shown in Table 3. The pelargonidin-3-(*p*-coumaroyl)digluoside-5-(malonyl)glucoside (peak 3) was 71.8% of the total anthocyanins peak area and decreased to 64.9% of total anthocyanins, whereas the pelargonidin-3-(*p*-coumaroyl) digluoside-5-glucoside (peak 1) increased slightly from 3.1% to 4.8% during the whole fermentation duration, suggesting that pelargonidin-3-(*p*-coumaroyl)digluoside-5-(malonyl)glucoside might degrade into pelargonidin-3-(*p*-coumaroyl)digluoside-5-glucoside by removal of a malonyl group. The percentage of pelargonidin-3-(feruloyl)diglucoside-5-(malonyl)glucoside increased from 19.5% to 23.6% due to the rates of release and degradation compared to other three anthocyanins. Pelargonidin-3-(feruloyl)diglucoside-5-glucoside did not change at the end of fermentation.

**Table 3:** Anthocyanins in radish brines during fermentation.

	Fermentation Duration (days)			
	2	5	9	14
Total Anthocyanins ^1^ (µg/mL)	19.30 ± 2.94a	18.23 ± 0.78a	18.43 ± 1.94a	15.35 ± 2.87a
Peak area % of individual anthocyanin ^2,3^				
Pg-3-(*p*-coumaroyl)diglu-5-glu	3.09 ± 0.24a	2.47 ± 0.27b	4.39 ± 0.40c	4.75 ± 0.36c
Pg-3-(feruloyl)diglu-5-glu	6.40 ± 0.39a	7.19 ± 0.22b	5.65 ± 0.23c	6.79 ± 0.21ab
Pg-3-(*p*-coumaroyl)diglu-5-(malonyl)glu	71.77 ± 0.57ab	70.25 ± 0.77ab	69.68 ± 0.98b	64.88 ± 1.28c
Pg-3-(feruloyl)diglu-5-(malonyl)glu	19.56 ± 1.12a	21.02 ± 0.54a	20.05 ± 0.77a	23.56 ± 0.57b

^1^ Calculation was based on 1 mL of brine as perlargonidin-3-glucoside equivalents. ^2^ Calculation was based on peak areas in HPLC chromatogram. ^3^ Calculation was based on percentage of peak areas in HPLC chromatogram. Abbreviation: Pg, pelargonidin; diglu, diglucoside; glu, glucoside. Values are represented as means ± SD (*n* = 3). Within each column, means with the same letter are not significantly different (*p* ≤ 0.05).

**Figure 3:**
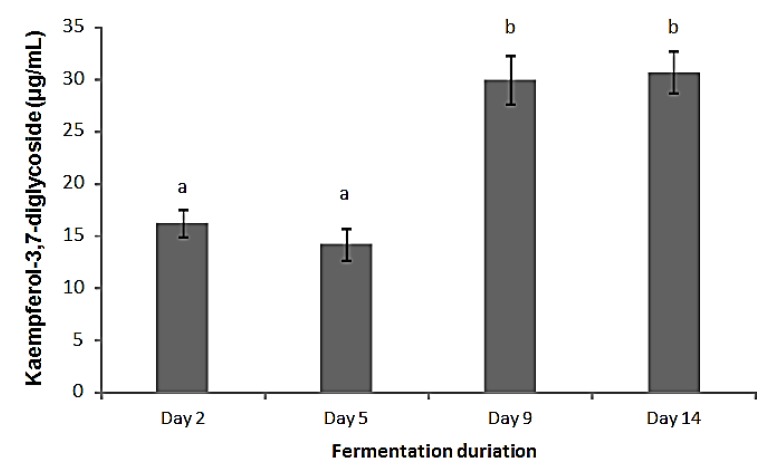
Changes in kaempferol-3,7-diglycoside levels in radish brines during fermentation. Results were expressed as µg kaempferol equivalents/mL. Tests were conducted in triplicate, with mean values shown and standard deviations depicted by the vertical bars. Columns marked with different letters are significantly different (*p* <0.05).

As shown in Figure 3, the kaempferol-3,7-diglycoside in radish brines during fermentation was calibrated as kaempferol equivalents using HPLC profiles. The amount of kaempferol-3,7-diglycoside was about 15 µg/mL in radish brines on the 2nd and 5th day of fermentation and it increased significantly to approximate 30 µg/mL on the 9th and 14th day, suggesting that kaempferol-3,7-diglycoside was released gradually from radish roots during fermentation and appeared to be more stable than anthocyanins. Additionally, the content of kaempferol glycoside in brines was considerably high compared to reports of 38.5 µg/g DW in white radishes [22].

### 2.4. Change in Total Phenolics and Phenolic Acids in Brines during Fermentation

About 208 µg gallic acid equivalents (GAE)/mL of total phenolics were released into the brine rapidly after two days of fermentation and the concentration remained consistent until the 5th day (Table 4). Total phenolics in radish brines were 220 µg GAE/mL and 206 µg GAE/mL on the 9th and the 14th day, respectively. However, changes in total phenolics during fermentation were not significant (*p* > 0.05).

**Table 4:** Total phenolics and free phenolic acids in radish brines during fermentation.

	Fermentation duration			
	Day 2	Day 5	Day 9	Day 14
Total phenolics ^1^ (µg/mL)	208.33 ± 9.81a	208.98 ± 13.61a	220.57 ± 11.59a	206.75 ± 5.08a
Free phenolic acids ^2^ (µg/mL)				
4-Hydroxybenzoic	19.41 ± 2.09a	18.51 ± 1.00a	15.81 ± 2.63a	11.31 ± 0.57b
Gentisic	10.93 ± 1.83ab	14.27 ± 2.57a	11.96 ± 1.06a	8.49 ± 0.60b
Vanillic	11.7 ± 0.74a	12.34 ± 0.86a	9.77 ± 1.51a	4.76 ± 0.34b
Syringic	8.61 ± 1.40a	8.1 ± 2.29a	2.31 ± 0.74b	2.96 ± 1.11b
*p*-Coumaric	1.54 ± 0.57a	1.16 ± 0.37a	2.06 ± 0.80a	0.51 ± 0.20b
Ferulic	3.86 ± 0.86a	4.89 ± 0.60ab	6.56 ± 1.26b	3.99 ± 0.34a
Sinapic	21.73 ± 1.34a	29.96 ± 2.03b	32.79 ± 2.77b	31.11 ± 3.14b
Salicylic	4.50 ± 1.29a	2.96 ± 1.11a	3.09 ± 1.17a	7.07 ± 1.09b
Total	82.28 ± 10.12ab	92.19 ± 10.83a	84.35 ± 11.94ab	70.2 ± 7.39b

^1^ Calculation was based on 1 mL of brine as gallic acid equivalents. ^2^ 4-hydroxybenzoic, gentisic, vanillic, syringic, *p*-cumaric, ferulic, sinapic, and salicylic stand for 4-hydroxybenzoic, gentisic, vanillic, syringic, *p*-coumaric, ferulic, sinapic and salicylic acids. Data are expressed as mean ± standard deviation (*n* = 3). Within each row, means with the same letter are not significantly different (*p* ≤ 0.05).

Phenolics including monomeric anthocyanins migrated from radish roots to brines, and reached to the equilibrium point during the initial two days of fermentation, which could partially explain how total phenolics in fermented vegetables usually decrease during pickling processes aside from degradation [19].

As shown in Table 4, 4-hydroxybenzoic, gentisic, vanillic, syringic, *p*-coumaric, ferulic, sinapic and salicylic acids were detected as free phenolic acids present in radish brines and in radish roots [14,23], although the caffeic acid reported by Stohr and Herrmann [23] and Mattila *et al*. [14] were not detected in this study. However, Mattila *et al*. found that only ferulic acid was present in radishes as free phenolic acid and others were conjugated or insoluble phenolic acids [14]. The occurrence of eight free phenolic acids, including ferulic acid, in radish brines could possibly be explained by the fact that conjugated and insoluble phenolic acids were hydrolyzed and released as free phenolic acids in brines during fermentation with *Lactobacillus plantarum*. The levels of free phenolic acids varied in brines during fermentation. 4-Hydroxybenzoic, gentisic, vanillic, syringic and sinapic acids were the predominant phenolic acids in brines, accounting for 88% (w/w) of the total free phenolic acids. 4-Hydroxybenzoic and syringic acid decreased from 19.41 µg/mL to 11.31 µg/mL and from 8.61 µg/mL to 2.96 µg/mL, respectively, whereas sinapic acid increased from 21.73 to 31.11µg/mL during the 14 day fermentation process. Both gentisic and vanillic acid increased slightly from the 2nd to the 5th day and then decreased with further fermentation. The total free phenolic acids were 82.28 µg/mL and 92.19 µg/mL on the 2nd and the 5th day, respectively, and then slightly but not significantly decreased on the 9th day, and dropped significantly to 70.2 µg/mL at the end of fermentation compared with the yield on the 5th day (*p* ≤ 0.05). The contents of total free phenolic acids in radish brines were comparable to the amount of total phenolic acid in radish roots of 120 µg/g FW reported by Mattila *et al*. [14].

### 2.5. Changes in Antioxidant Activity of Brines during Fermentation

To further investigate the nutritional value of the fermented red radishes, the antioxidant activity of the extracts were determined by the oxygen radical absorbance capacity (ORAC), the ability to chelate Fe^2+^, and the ability to reduce Fe^3+^-TPTZ to a less reactive form (FRAP) tests.

**Figure 4:**
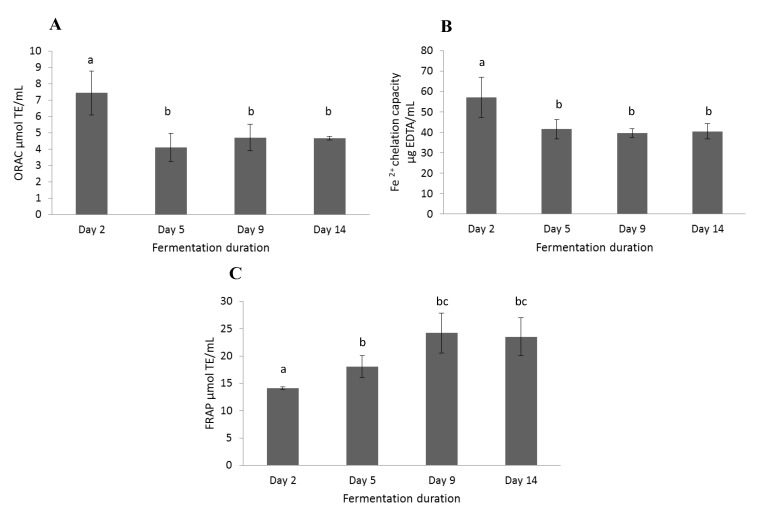
Analysis of the antioxidant capacity of radish brines during fermentation. (**A**) ORAC of radish brines expressed as µmol Trolox equivalents/mL; (**B**) Fe^2+^ chelation capacity of radish brines expressed as µg EDTA equivalents/mL; (**C**) FRAP of radish brines expressed as µm Trolox equivalents/mL. Tests were conducted in triplicate, with mean values shown and standard deviations depicted by the vertical bars. Columns marked with different letters are significantly different (*p* < 0.05).

All radish brines during the fermentation showed antioxidant activity under the experimental conditions as shown in Figure 4. The brine obtained on the 2nd day of radish fermentation had an ORAC value of approximate 7.5 μm TE/mL and the Fe^2+^ chelation capacity of approximate 60 μg EDTA/mL, that decreased substantially to 4–5 μm TE/mL and approximate 40 μg EDTA/mL, respectively, at the 5th, 9th, and 14th day of fermentation as shown in Figure 4A and 4B. The reducing power of radish brines in Figure 4C increased from approximate 14 μm TE/mL on the 2nd day to approximate 24 μm TE/mL on the 9th and 14th day using the FRAP test. All results implied that the radish brine after fermentation with *Lactobacillus plantarum* was rich in anthocyanin-type pigments, kaempferol derivatives and phenolic acids, and exhibited high antioxidant ability according to its oxygen radical absorbance, metal chelation, and reducing power. Therefore radish brine might be used as a highly valuable byproduct as a source of natural pigments or nutraceuaticals instead of just being an industrial waste.

## 3. Experimental

### 3.1. Materials and Chemicals

Radishes (*Raphanus sativus* L.) were purchased from a local grocery (Shanghai, China). Starter culture of *Lactobacillus plantarum* was kindly donated by Professor Dong, Jiangsu University, China. Phenolic acid standards including gallic, 4-hydroxybenzoic, gentisic, vanillic, syringic, *p*-coumaric, ferulic, and sinapic acids, and kaemferol were purchased from Aladdin Company (Shanghai, China). All other chemicals were purchased from Sigma-Aldrich (Shanghai, China).

### 3.2. Fermentation

The fermentation of radish peels was performed according to the literature [24]*.* Radish peel was cleaned and cut into 1 cm × 2 cm. The radish peels were heated for 15 min at 100 °C and immediately cooled down to room temperature. About 15 glass jars were added with 30 g of blanched radish peels, 60 mL of 8% NaCl, respectively, inoculated with 5 mL of 1 × 10^7^ CFU/mL starter culture and sealed. All jars were kept in dark at 25 °C for 2 weeks and three jars were sampled randomly at intervals during the fermentation for further analyses. The day 0 was the time right before radish peels was added with brines. The day 2 was the time when the when the brine and radish root peels mixed and kept in dark for 24 h, and so on.

### 3.3. Lactic Acid

The brine was filtered through 0.45 µm hydrophilic membranes (Anpel, Shanghai, China) and analyzed for the lactic acid in an Agilent 1260 HPLC system using a Zorbax SB-Aq column (150 × 4.6 mm, 5 μm, Agilent Technologies, Palo Alto, CA, USA) fitted with a Zorbax SB-Aq guard cartridge (4.6 × 12.5 mm, 5 μm). The lactic acid was separated using a gradient elution program with a mobile phase containing solvent A (formic acid/methanol/H_2_O, 0.1:3:96.9, v/v/v) and solvent B (acetonitrile). Separation was achieved through a gradient elution, as following: 100% A, 0–5 min; 100% to 50% A, 5–20 min. An injection volume of 30 μL with 0.8 mL/min flow rate was used. The data were collected at 210 nm. Lactic acid content in radish brine was calculated using external standard. Each sample was analyzed in triplicates.

### 3.4. pH and Colour Properties

Brine samples were tested for their pH values and colour properties. Hue angle, chroma, and lightness were measured with a Hunter ColorQuest XE colorimeter (HunterLab, Reston, VA, USA) using illuminant C and 10° observer angle. Brine samples after pH measurement were placed in 1 cm path length disposable cuvettes and read using the reflectance specular included mode and covered with light trap using total transmittance. Three replicates were performed.

### 3.5. Total Phenolics

Total phenolics were measured using a modified Folin-Ciocalteu method described by [25]. A series of tubes were prepared with 15 mL of water and 1 mL of Folin-Ciocalteu reagent. Then, 1 mL of brine samples, gallic acid dilutions (standards), and water blank was added into tubes, mixed well, and left to stand at room temperature for 10 min. 20% (w/v) Na_2_CO_3_ solution (3 mL) was added to each test tube and mixed well before they were put at ambient temperature for 2 h reaction. After incubation, tubes were immediately cooled down in an ice bath. The absorbance of samples and standards was measured at 765 nm using a L5S UV-visible spectrophotometer (Shanghai Analytical Instrument, Shanghai, China). Total phenolics were calculated as gallic acid equivalents based on a gallic acid standard curve.

### 3.6. Total Monomeric Anthocyanins

The total monomeric anthocyanin content was measured by the pH differential method [26]. A L5S UV-visible spectrophotometer was used to read absorbance at the maximum visible wavelength of absorption of each extract (ranging from 490 to 535 nm) and at 700 nm. Monomeric anthocyanins were calculated as equivalents of pelargonidin-3-glucoside, using the extinction coefficient of 31,600 L cm^−1^ mg^−1^ and a molecular weight of 433.2 g L^−1^ [2]. Cuvettes of 1 cm path length were used. Analyses were performed in triplication per treatment.

### 3.7. Analytical Chromatography of Anthocyanins and Kaempferol-3,7-Diglucoside in Radish Brines

Radish brines (2 mL) from each cultivar were semi-purified using the method described in [27] before MS and HPLC analyses. About 1 mL of anthocyanin extract was loaded onto a C18 Sep-Pak solid cartridge (ANPEL Scientific Instrument, Shanghai, China), which was preconditioned with 2 column volumes of methanol and 3 column volumes of 0.01%-HCl-acidified water. Anthocyanins and other flavonoids were bound to the C18 cartridge, whereas sugars and other polar compounds were removed with 3 column volumes of 0.01%-HCl-acidified water. Flavonoids including anthocyanins were recovered from the cartridge with three column volumes of 0.01%-HCl-acidified methanol. The methanol was removed by rotary evaporation at 40 °C, and the residue was taken up to about 1 ml with deionized water. The sample from the 0.01%-HCl-acidified methanol fraction was stored at about 18 °C for analysis.

Anthocyanins and other flavonoids were qualitatively analyzed using a UPLC-HRMS system consisting of a Waters Micromass Q-TOF Premier mass spectrometer equipped with an electrospray interface (Waters Corporation, Milford, MA, USA) located at the Instrumental Analysis Center of Shanghai Jiao Tong University. The positive ionization and negative ionization modes were used for anthocyanins and other flavonoids, respectively. The applied electrospray/ion optics parameters were set as follows: capillary voltage, 3.0 kV (positive mode) and 2.6 kV (negative mode); sampling cone, 35 V (positive mode) and 55 V (negative mode); collision energy, 4 eV; source temperature, 100 °C; desolvation temperature, 300 °C; desolvation gas, 500 L/h. Spectra were collected using full ion scan mode over the mass-to-charge (*m/z*) range 200–2000. Scan time, 0.3 s; interscan time, 0.02 s.

Quantitative analyses of anthocyanins and other flavonoids were performed using the Agilent 1100 system equipped with a photo-diode-array detector. Separation were achieved by reverse phase elution on a 5 μm Shim-pack VP-ODS column (4.6 mm × 250 mm, Shimadzu, Kyoto, Japan) fitted with a 4.6 × 10 mm Shim-pack GVP-ODS guard column (Shimadzu). Solvents and sample were filtered through 0.45 µm hydrophobic/hydrophilic membranes (Shanghai Yaxing Corp, Shanghai, China) and 0.45 µm nylon membrane filters (Shanghai Mosu Scientific Instruments and Materials, Shanghai, China), respectively. The chromatographic conditions were: flow rate 1 mL/min, sample injection volume of 10 µL and mobile phase A (formic acid/water, 0.1:99.9, v/v) and mobile phase B (formic acid/acetonitrile, 0.1:99.9, v/v). A gradient program was used as follows: 0–5 min, 3% B; 5–10 min: from 3% to 15% B; 10–30 min, from 15% to 25% B; 30–35 min, from 25% to 45%; 35–45 min. Spectral information over the wavelength range of 210–600 nm was collected.

### 3.8. Phenolic Acids

The free phenolic acids in the radish brines were determined quantitatively by a literature HPLC method [28]. The free phenolic acids in brines were adjusted pH to 2–3 and then extracted in ethyl acetate and ethyl ether (1:1, v/v). After evaporation of ethyl acetate and ethyl ether, each phenolic acid extract was quantitatively redissolved in MeOH and analyzed by Agilent 1100 HPLC system using a 5 μm Shim-pack VP-ODS column (4.6 mm × 250 mm, Shimadzu) fitted with a 4.6 × 10 mm Shim-pack GVP-ODS guard column (Shimadzu). Phenolic acids were separated using a gradient elution program with a mobile phase containing solvent A (formic acid/H_2_O, 0.1:99.9, v/v) and solvent B (formic acid/acetonitrile, 0.1:99.9, v/v). Separation was achieved through a gradient elution, as following: 7% B, 0–5 min; a linear gradient from 7% to 25% B, 5–45 min; 25% to 45% B, 45–55 min. An injection volume of 10 μL with 1 mL/min flow rate was used. Identification of phenolic acids was accomplished by comparing the retention time and spectrum of peaks in the samples to that of the standards under the same HPLC conditions detected at 280 nm wavelength. Quantification of each phenolic acid was determined using external standards and total area under each peak. All analyses were carried out in triplicate.

### 3.9. Antioxidant Activity Assays

#### 3.9.1. Oxygen Radical Absorbance Capacity (ORAC) Assay

The determination of oxygen radical absorbing capacity of the extracts from the fermented red radish was performed according to the previously reported procedure [29] in an Infinite F200 PRO microplate reader (Tecan, Männedorf, Switzerland). Samples and Trolox standards were prepared with 50% acetone. All other reagents were prepared in 75 mmol/L phosphate buffer (pH 7.4). Briefly, each well in 96-well plate contained 30 μL sample or 50% acetone for blank, and 225 μL fluorescein (81.63 nmol/L). The plate with cover was incubated for 20 min in 37 °C, and then 25 μL AAPH (0.36 mol/L) were added to each well to start reaction, resulting in a final total volume of 280 µL. The fluorescence was recorded every 5 min for 1 h (ex/em: 485/538 nm) at 37 °C. Standards and samples were performed in triplicate. Trolox equivalents were calculated using the relative area under the curve for samples compared to a Trolox standard curve prepared under the same experimental conditions. Reactions were conducted in triplicate and results are expressed as micromoles of Trolox equivalents per milliliter of brines.

#### 3.9.2. The Ferric Reducing Ability of Plasma (FRAP) Assay

The FRAP assay was determined based on the reduction of Fe^3+^-TPTZ to a blue coloured Fe^2+^-TPTZ with modification [28]. The FRAP reagent was prepared by mixing 300 mmol/L acetate buffer (pH 3.6), 10 mmol/L TPTZ and 20 mmol/L FeCl_3_·6H_2_O in a ratio of 10:1:1 (v/v/v). Then, 3 mL of FRAP reagent, 100 μL of sample or standards and 300 μL of distilled water were added to the test tubes, and incubated at 37 °C for 30 min. Absorbance was measured at 590 nm using an Infinite F200 PRO microplate reader (Tecan). Trolox was used as standard for comparison and adequate dilution of sample was performed. Reactions were conducted in triplicate and results were reported as micromoles of Trolox equivalents (TE) per milliliter of radish brine.

#### 3.9.3. Fe^2+^ Chelating Ability

Fe^2+^-chelating ability of the extract was determined according to the previous [30]. The Fe^2+^ level was monitored by measuring the formation of the ferrous ion-ferrozine complex. The brine (1.0 mL) was mixed with 3.7 mL methanol, 0.1 mL of 2 mmol/L FeCl_2_ and 0.2 mL of 5 mmol/L ferrozine. The mixture was left at room temperature for 10 min. The absorbance of the resulting solution was measured at 565 nm. EDTA was used as standard for comparison and adequate dilution of sample was performed. Reactions were conducted in triplicate and results were reported as micrograms of EDTA equivalents per milliliter of brine.

### 3.10. Statistical Analysis

Tests were conducted in triplicate determinations with data reported as mean ± standard deviation. One-way ANOVA and LSD test at the level of 0.05 were used to identify differences in means. Statistics were analyzed using SPSS for Windows (version rel. 10.05, 1999, SPSS Inc., Chicago, IL, USA).

## 4. Conclusions

The radish brines after a two-week fermentation of red radishes with *Lactobacillus plantarum* were rich in the lactic acid about 6.23 g/L and presented a bright red colour (Lightness = approximate 71, Chroma = approximate 43, Hue = approximate 27), attributable to the presence of considerable anthocyanins released from radish roots. Many phytochemicals were released from roots to brines during the fermentation. Four anthocyanins were detected in brines and characterized as pelargonidin-3-diglucoside-5-glucoside derivatives acylated with one coumaric acid, one ferulic acid, or both. The kaempferol-3,7-diglycoside was increasing with fermentation. Eight kinds of free phenolic acids were detected in radish brines, suggesting that the fermentation encouraged free phenolic acid release from conjugated or insoluble forms. Generally the content of total phenolics (206 to 208 μg/mL) did not change significantly during fermentation. The oxygen radical absorbance capacity (ORAC) and Fe^2+^ chelating ability of radish brines appeared to decrease whereas the reducing power (FRAP) tended to increase during the fermentation.

The radish brines that have usually been discarded as wastes are therefore a potentially valuable byproduct for their colour and antioxidative properties. However, the duration of fermentation of radish brines should be considered for pigments, phytochemicals and antioxidant activities. It is noted that most of phytochemicals and the antioxidant activities of radish brines reached their highest levels by the 5th and 9th day of fermentation than other days, presumably because many phytochemicals would be released less or be degraded more with a shorter or longer fermentation.
